# Molecular identification of cestodes from rodents in the Mazury Lake District region of Poland

**DOI:** 10.1007/s00436-026-08629-x

**Published:** 2026-02-16

**Authors:** Wiktoria Romanek, Mohammed Alsarraf, Mustafa Alsarraf, Dagmara Wężyk, Dorota Dwużnik-Szarek, Aleksander Goll, Joanna Górska, Martyna Krupińska, Maciej Grzybek, Katarzyna Tołkacz, Jolanta Behnke-Borowczyk, Jerzy M. Behnke, Anna Bajer

**Affiliations:** 1 Department of Eco-Epidemiology of Parasitic Diseases, Institute of Developmental Biology and Biomedical Sciences, Faculty of Biology, University of Warsaw, Warsaw, Poland; 2 Department of Microbiology, Molecular Genetics and Genomics, Centre for Advanced Materials and Technologies (CEZAMAT), Warsaw University of Technology, 19 Poleczki St, 02-822 Warsaw, Poland; 3 Department of Tropical Parasitology, Institute of Maritime and Tropical Medicine, Medical University of Gdańsk, Gdynia, Poland; 4 Institute of Biochemistry and Biophysics, Polish Academy of Sciences, Pawińskiego 5A, 02-106 Warsaw, Poland; 5 Department of Forest Phytopathology, Faculty of Forestry, Poznań University of Life Sciences, Poznań, Poland; 6 School of Life Sciences, University of Nottingham, University Park, Nottingham, NG7 2RD UK

**Keywords:** *Paranoplocephala*, *Catenotaenia*, *Skrjabinotaenia*, *Spasskijela*, *Mesocestoides*, Phylogenetic analysis

## Abstract

Rodents serve as hosts for many species of parasites, including cestodes, but the taxonomy of rodent-infecting tapeworms, especially in Central Europe, is still understudied. Therefore, the current study was conducted to identify larval and adult cestodes in free-living rodents from Northeastern Poland. The fieldwork was conducted between 2014 and 2023 in three forest sites. Cestodes were collected from five rodent species (*Clethrionomys glareolus, Alexandromys oeconomus*, *Microtus agrestis, Apodemus agrarius* and *A. flavicollis*, and were identified through morphology and by several genetic markers (12S rDNA, *cox1, nad 1*, 28S rDNA*,* 12S-16S rDNA) using phylogenetic analyses. A total of 58 cestode specimens were analyzed using molecular and phylogenetic analyses. Species identified included *Catenotaenia henttoneni, Spasskijela kratochvili, Mesocestoides litteratus, Mesocestoides melesi*, and others. Three *Paranoplocephala* specimens were identified as *P. kalelai* based on *nad1* sequences. Morphological and genetic analyses indicated that the dominant adult cestode in bank vole was *C. henttoneni* and *Mesocestoides* spp. were the dominant larval cestodes. This study combined genetic and morphological methods to identify the cestodes infecting rodents in Poland. It confirmed the existence of *P. kalelai* in Poland, suggesting that previously reported *Paranoplocephala* spp., identified by morphological characters, may have corresponded to this molecularly identified species. Our study also highlighted gaps in genetic databases that currently still do not allow precise species identification.

**Supplementary Information:**

The online version contains supplementary material available at 10.1007/s00436-026-08629-x.

## Background

The structure of intestinal parasite communities of rodents has been well studied in Europe in recent years (Behnke et al. 1999; 2001; 2022; 2024). Identification of helminths was historically primarily based on morphological features and until recently, genetic data have been limited for rodent-infecting helminths, i.e. nematodes (Stewart et al. 2018; Behnke et al. 2022) or cestodes (Haukisalmi and Tenora 1993; Haukisalmi et al. 2014; Bajer et al. 2020). The availability of modern genotyping tools, encompassing a range of genetic markers, including mitochondrial (mt) and nuclear genes/regions (i.e. 12S and 28S rDNA, mt cytochrome c oxidase subunit 1 [*cox1*] or NADH dehydrogenase subunit 1 [*nad1*]) and genetic databases allows an improved understanding of parasite phylogeny/evolution. Moreover, molecular tools also provide a means for the identification of cryptic species, that cannot be easily distinguished by conventional morphology. Progress in this area has resulted in numerous changes in our understanding of parasite taxonomy and consequently to the keys used for identification of species. A good example is the recent description of 12 new genera of tapeworms from the so called ‘*Paranoplocephala *complex’ (Haukisalmi et al. 2014). We have also recently reported on two species of *Mesocestoides* larvae parasitizing small rodents in NE Poland: *Mesocestoides litteratus* and *Mesocestoides melesi* (Bajer et al. 2020).

The main aim of our study was to determine the genetic identity of larval and adult cestodes parasitizing free-living rodents in the Mazury Lake District region of Poland. In our study we combined the phylogenetic analyses of several genetic markers with morphological features for cestode identification. Particular focus was on 1) genotyping of tapeworms from the *Paranoplocephala* complex parasitizing bank voles (*Clethrionomys glareolus*) and 2) on the genetic identification of catenotaeniid cestodes, following the recent update of the molecular systematics of this cestode family (Haukisalmi et al. 2018, 2014).

## Material and methods

### Study sites

Larval and adult cestodes were acquired between 2014 and 2023, during our long-term studies on rodent helminths in the Mazury Lake District in the Northeastern corner of Poland, in three forest areas in the vicinity of Jezioro (Lake) Śniardwy. Site 1 is referred to as Urwitałt (N 53 × 48.153, EO 21 × 39.784), Site 2 as Tałty (N 53 × 53.644, EO 21 × 33.049) and Site 3 as Pilchy (N 53 × 42.228, EO 21 × 48.499) after nearby settlements (Additional file 2: Table S1). Additionally, *Microtus* spp. were trapped in fallow lands near the field station in Urwitałt. The study sites have been described comprehensively by Behnke et al. (2008a, b; 2001) and Tołkacz et al. (2017).

The methods used for trapping rodents, and for sampling and processing trapped animals, including the lists of appropriate permits, have all been provided in our previous papers (Behnke et al. 2001; 2008a, b; Grzybek et al. 2015; Tołkacz et al. 2017). Briefly, animals were live-trapped, transported to a laboratory in field station (Urwitałt), sacrificed by overexposure to Isofluran anaesthetic and subsequent cervical dislocation. Autopsies were carried out within 1–2 h following the death of an animal. Larval cestodes were collected from body and pleural cavities, and also after dissection of the liver. Adult tapeworms were collected either immediately after extraction from a section of the small intestine or after 2 h of incubation in 0.9% saline in Petri dishes. Dish content was examined under a stereo microscope. The collected specimens (larvae and adults) were kept in saline until fixation: distal parts of larvae (large larvae) or whole specimen (*Mescocestoides*) and several mature proglottids of adult tapeworms were placed in 70% ethanol for molecular study, while other parts of tapeworms were fixed in AFA solution (100 ml 40% formaldehyde, 250 ml 95% ethanol, 100 ml glycerine, 50 ml glacial acetic acid, 500 ml distilled water) after flattening between two slides. Following incubation in AFA for 24 h, specimens were carefully transferred to 70% ethanol filled vials and transported to our laboratory in Warsaw.

### Nomenclature

In this paper we refer to *Clethrionomys glareolus* (as recommended by the Mammal Diversity Database, Tesakov et al. 2010 and Kryštufek et al. 2020), rather than *Myodes glareolus* (see Carleton et al. 2014), following several recent revisions of the generic attribution of bank voles and to *Craseomys rufocanus* Sundevall, 1846 rather than *Clethrionomys* or *Myodes rufocanus* (See Abramson and Lissovsky 2012).

We refer also to *Spasskijela kratochvili* rather than *Skrjabinotaenia lobata* following Haukisalmi et al. (2018) and Haukisalmi and Elmahy (2025). Although Haukisalmi et al (2018) restored *Spasskijela lobata* (Baer 1925) Tenora 1959 for *Skrjabinotaenia lobata*-like species A, B and C, in clade 11 of the phylogenetic tree based on concatenated sequences of 18S, 23S-16S rDNA, these authors then suggested that isolates from European rodents (Species C) should be referred to as *Spasskijela kratochvili* Tenora 1959.

### Morphological examination of larval and adult tapeworms

Larval and adult tapeworms were stained using borax carmine, dehydrated in an ethanol series and mounted in Canada balsam for microscopical examination. Specimens were inspected carefully, using a Zeiss STEMI 508 stereo microscope (Germany) equipped with a camera and identified initially based on morphologic characters (Additional file 1: Supplementary Fig. 1).

### DNA extraction and amplification

Genomic DNA was extracted from specimens fixed in 70% EtOH using about 20 mg of tissue, with the DNAeasy Blood & Tissue kit (Qiagen, Hilden, Germany) and stored at a temperature of −20°C.

Molecular typing of tapeworms was performed by amplification and sequencing of several genetic markers. A 350 bp-fragment of mitochondrial (mt) 12S rDNA was amplified using the primers P60 for (5′-TTA AGA TAT ATG TGG TAC AGG ATT AGA TAC CC – 3′) and P375 rev (5′-AAC CGA GGG TGA CGG GCG GTG TGT ACC – 3′) (Von Nickisch-Rosenegk et al. 1999). A 400 bp fragment of the cytochrome c oxidase subunit 1 (*cox1*) was amplified using the primers JB3 (5′-TTT TTT GGG CAT CCT GAG GTT TAT—3′) and JB45 (5′-TAA AGA AAG AAC ATA ATG AAA ATG-3′) (Kumar et al. 2018).

To identify new species of the *Paranoplocephala* complex, an 850 bp fragment of the dehydrogenase subunit 1 (*nad1*) was amplified using the primers NAD1F (5′- GGN TAT TST CAR TNT CGT AAG GG −3 ′) and trnNR (5′- TTC YTG AAG TTA ACA GCA TCA - 3′) (Haukisalmi et al. 2014).

For molecular typing of *Catenotaenia* and *Spasskijela*, the partial 28S rDNA (1,500 bp) and mitochondrial 12S–16S rRNA (820 bp) genes were amplified (Haukisalmi et al. 2018). The 12S–16S rDNA region encompasses three genes: regions coding for the large subunit ribosomal RNA (400 bp), tRNA-Cys gene (76 bp) and small subunit ribosomal RNA (345 bp) (Haukisalmi et al. 2018). A 28S rDNA fragment was amplified using the primers XZ-1 F (5′ ACCCGCTGAATTTAAGCATAT 3′) ((Waeschenbach et al. 2007) and 1500R (5′ GCTATCCTGAGGGAAACTTCG 3′) (Littlewood et al. 2008). The 12S–16S fragment was amplified using the primers Hym-16S-F (5′ TTATAAATGGCCGCAGTATATTGAC 3′) and Hym-12S-R (5′ ATCGTCCTTTATAACACACCTTCCC 3′) (Von Nickisch-Rosenegk et al. 2001).

The PCR reactions were performed in a volume of 20 μl, including 10 × PCR Dream Taq Green buffer (Thermo Fisher Scientific, Waltham, Massachusetts, USA), 5U Dream Taq polymerase (Thermo Fisher Scientific), 0.33 mM dNTPs, 1 μM of each primer and 2 μl of the extracted DNA sample. Negative controls were performed with nuclease-free distilled water, in the absence of template DNA.

PCR reactions for 12S rDNA and *cox1* were carried out under identical cycling conditions: primary denaturation at 94 °C for 3 min, followed by 40 cycles of denaturation at 94 °C for 30 s, annealing at 56 °C for 1 min, and elongation at 72 °C for 1 min, followed by a final elongation step at 72 °C for 7 min and a hold step at 4 °C.

PCR reactions for *nad1* were carried out under the following cycling conditions: primary denaturation at 94 °C for 3 min, followed by 40 cycles of denaturation at 94 °C for 30 s, annealing at 55 °C for 30 s, and elongation at 72 °C for 1.5 min, followed by a final elongation step at 72 °C for 7 min and a hold step at 4 °C.

PCR reactions for 28S rDNA and mitochondrial 12S–16S rDNA were: primary denaturation at 94 °C for 3 min, followed by 40 cycles of denaturation at 94 °C for 45 s, annealing at 55 °C for 45 s, and elongation at 72 °C for 45 s, followed by a final elongation step at 72 °C for 7 min and a hold step at 4 °C.

PCR products were subjected to electrophoresis on a 1.5% agarose gel and stained with Midori Green stain (Nippon Genetics, Düren, Germany). PCR products were sequenced in both directions using Sanger method by a private company (Genomed S.A., Warsaw, Poland) with the primers used for DNA amplification. The obtained sequences of each sample were quality checked, trimmed, and then the consensus sequence was constructed using Molecular Evolutionary Genetics Analysis version 11 (MEGA v. 11) (Tamura et al. 2001).

### Phylogenetic analyses

All sequences for each marker were checked in BLAST to identify the donor species. For each marker all obtained sequences were aligned with reference sequences from GenBank using the ClustalW in MEGA v.11, and AliView (Larsson 2014). Phylogenetic relationships among the 12S rDNA, *cox1* and *nad1* genes sequences were performed separately. Representative samples of cestodes were assessed using Bayesian Inference, implemented in MrBayes v3.2.6. (Ronquist et al. 2012). Due to the large size of the dataset, we fitted a GTR + G model with all six rate parameters free and with variation in the rate of evolution among sites in the alignment. Four independent runs of 10 000 000 generations were sampled every 1000 generations and 25% of the initial samples were discarded as a burn-in phase. The convergence of runs and effective sample sizes for the model’s parameters were checked using Tracer v. 1.7 (Rambaut et al. 2018).

For the phylogenetic analysis of catenotaeniids two genetic markers were analysed: a ~ 950 bp fragment of the 12S-16S rDNA and a 1600 bp fragment of the 28S rDNA (Additional file 3: Table S2). All obtained DNA sequences were submitted to GenBank (Additional file 2: Table S1). For each cestode, the sequences of the two genes were merged into a single ~ 2600 bp long sequence.

## Results

In total 58 cestode specimens were examined initially by morphological characters and then molecular methods based on a combination of several genetic markers (*cox1*, 12S rDNA, *nad1,* 28S rDNA*,* 12S-16S rDNA) from five rodent species: 49 specimens from *C. glareolus*, one from *M. oeconomus*, one from *M. agrestis,* one from *A. agrarius* and six from *A. flavicollis* (Additional file 2: Table S1). Twenty specimens represented larval cestodes, derived from the body cavities or livers of their hosts, while 38 were adult tapeworms.

**Fig. 1:**
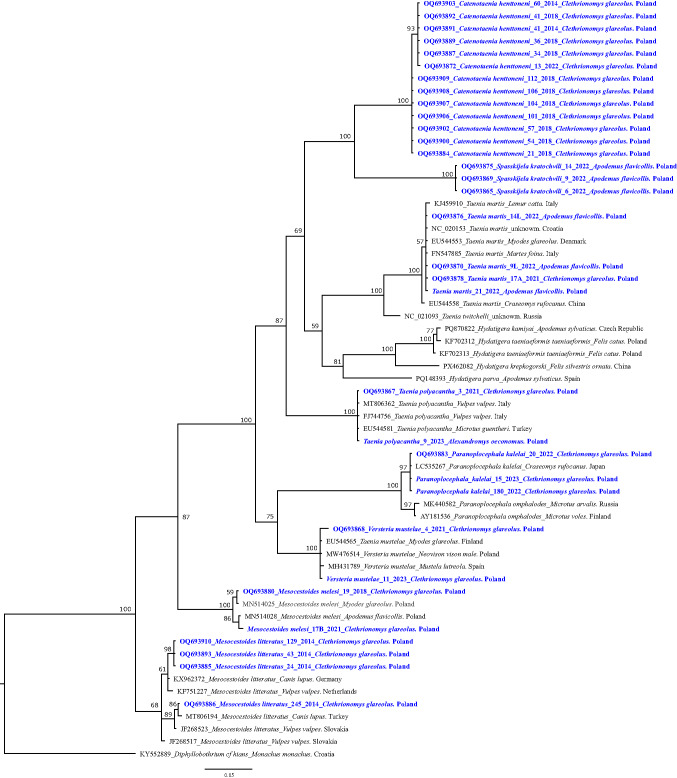
The phylogenetic tree of cestodes inferred from sequence variation of ~ 370 bp *cox1* gene fragment (*n* = 61). The tree is 50%-majority rule consensus obtained using MrBayes (Bayesian Inference). Numbers along nodes represent *a posteriori* probability. The DNA sequences obtained in this study are coloured in blue and bolded (*n* = 33)

**Fig. 2:**
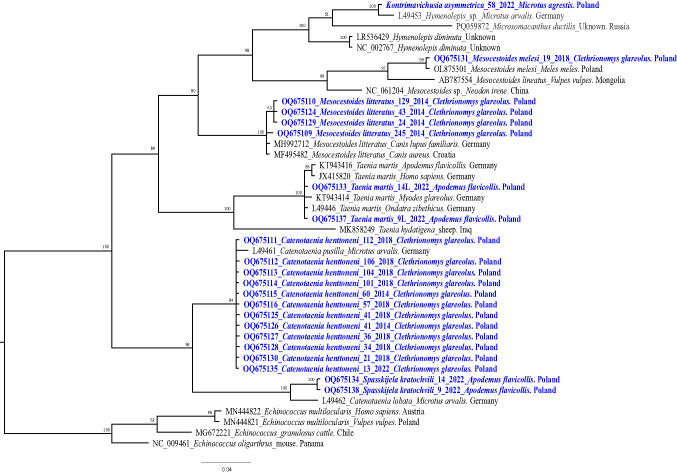
The phylogenetic tree of cestodes inferred from sequence variation of ~ 270 bp 12S rDNA gene fragment (*n* = 42). The tree is 50%-majority rule consensus obtained using MrBayes (Bayesian Inference). Numbers along nodes represent *a posteriori* probability. The DNA sequences obtained in this study are coloured in blue and bolded (*n* = 22)

**Fig. 3:**
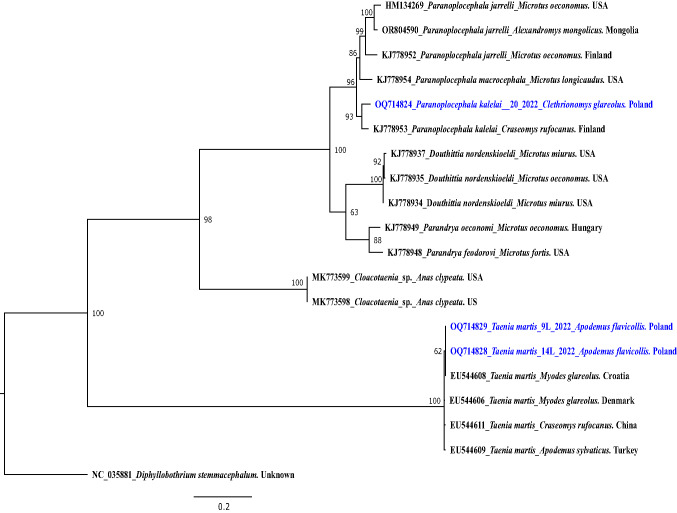
The phylogenetic tree of cestodes inferred from sequence variation of ~ 720 bp *nad1* gene fragment (*n* = 20). The tree is 50%-majority rule consensus obtained using MrBayes (Bayesian Inference). Numbers along nodes represent *a posteriori* probability. The DNA sequences obtained in this study are coloured in blue and bolded (*n* = 3)

**Fig. 4:**
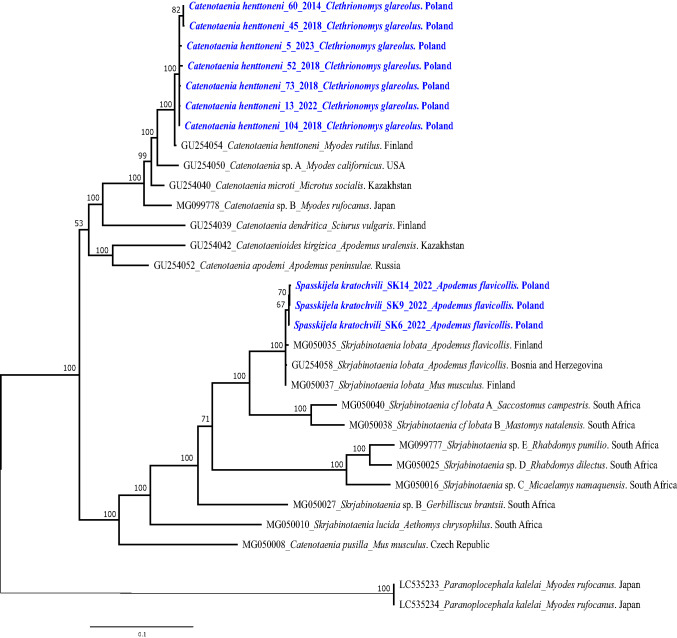
The phylogenetic tree of cestodes inferred from sequence variation of ~ 2900 bp concenated 12S-16S and 28S rDNA fragments (*n* = 31). The tree is 50%-majority rule consensus obtained using MrBayes (Bayesian Inference). Numbers along nodes represent *a posteriori* probability. The DNA sequences obtained in this study are coloured in blue and bolded (*n* = 10)

Preliminary microscopical examination of morphological features evident on stained slides (Additional file 1: Fig. S1), followed by molecular typing revealed that among the 58 specimens, 31 obtained from bank voles were *C. henttoneni* (Additional file 2: Table S1, Figs. 1, 2, 3 and 4) and the remaining specimens were *Taenia polyacantha* larvae (3 specimens), *Versteria mustelae* larvae (2 specimens), *Spasskijela kratochvili* adults (3 specimens), *Mesocestoides litteratus* larvae (7 specimens), *T. martis* larvae (5 specimens), *M. melesi* larvae (3 specimens), *Paranoplocephala kalelai* adults (3 specimens) and *Kontrimavichusia asymetrica* adult (1 specimen) (Additional file 2: Table S1, Figs. 1, 2, 3 and 4).

Larvae of *T. polyacantha,* collected from the pleural and peritoneal cavities of *A. agrarius, M. oeconomus* and *C. glareolus* showed 100% identity of *cox1* sequence with *T. polyacantha* sequences deposited in GenBank (*T. polyacantha* from *Vulpes vulpes,* Italy, MT806362) and 100% identity of 12S rDNA with several sequences of *T. polyacantha* (for example *T. polyacantha* from *V. vulpes*, Zürich, MZ414196). Our sequences clustered on one branch with *T. polyacantha* sequences obtained from red foxes in Italy (Fig. 1).

Larvae of *V. mustelae* from the livers of *C. glareolus* were identified based on 99.1% similarity of the *cox1* sequence with the *V. mustelae* sequence from the GenBank (*V. mustelae* from *Neovison vison*, Poland, MW476516). On the phylogenetic tree, our *V. mustelae* sequences grouped with *V. mustelae* sequences from the American mink from Spain and Poland (Fig. 1).

The larvae of *Mesocestoides* spp. cannot be distinguished morphologically but the three species of the *Mesocestoides* spp. known to infect rodents in Europe live in the peritoneal and pleural cavities, and liver surfaces of *C. glareolus.* Among 10 samples of *Mesocestoides* larvae, seven *cox1* sequences showed 100% identity to *M. litteratus* sequences from our previous study (*M. litteratus* from *A. flavicollis*, Poland, MN514033) (Bajer et al. 2020). On the phylogenetic tree, our seven *M. litteratus* sequences clustered with the *M. litteratus* sequences obtained from a golden jackal from Croatia and a dog from Germany (12S rDNA, Fig. 2). The remaining three *Mesocestoides* sequences showed 100% similarity to *M. melesi* from *C. glareolus* from Poland (MN514025).

Larvae of *T. martis* were collected from the pleural and peritoneal cavities of *A. flavicollis* and were identified based on 100% sequence identity of three markers with sequences from GenBank: including 12S rDNA (*T. martis* from *Homo sapiens*, Germany, LT837855), *cox1* (*T. martis* from *C. glareolus*, Denmark, EU544553) and *nad1* (*T. martis*, Croatia, NC020153). Sequences of *T. martis* from this study grouped with *T. martis* sequences from *C. glareolus, A. flavicollis* and *H. sapiens* from Germany (Fig. 2).

For *C. henttoneni* the highest percentage similarity of the *cox 1* gene was only 92%, and this to an unidentified *Catenotaenia* sp. (*Catenotaenia* sp. from *Craseomys rufocanu*s, Japan, LC535283). More specific identification based on the *cox 1* gene was not possible because unfortunately, as of to-date no *C. henttoneni cox1* sequences have been deposited in the GenBank database. Our phylogenetic analyses based on two genetic markers, *cox1* and 12S rDNA, showed that our specimens, formed a well-defined clade, closely related to *S. cf. lobata* (Figs. 1 and 2). In the phylogenetic tree based on combined 28S rDNA and 12S-16S rDNA (Fig. 4) our *C. henttoneni* sequences formed a well-supported sister clade to *C. henttoneni* isolated from *Clethrionomys rutilus* (northern red-backed voles)*,* Finland (GU254054 + MG049995), and differed from this sequence by 1–2 SNPs, hence most likely representing a *C. henttoneni* genotype/variant adapted to bank voles, rather than northern red-backed voles, or a geographical variant of this species.

Our *S. kratochvili* 12S *rDNA* sequences displayed the highest similarity of 96% to *Catenotaenia* (*Skrjabinotaenia) lobata* from *M. arvalis,* Germany (L49462). Phylogenetic analysis of 12S rDNA revealed that *S. kratochvili* from *A. flavicollis* formed a separate branch on the tree with *Catenotaenia (Skjabinotaenia) lobata* from *M. arvalis* from Germany (Von Nickisch-Rosenegk et al. 1999) (Fig. 2). In the phylogenetic tree for catenotaeniids based on combined 28S rDNA and 12S-16S rDNA (Fig. 4) our *S. kratochvili* sequences formed a well-supported clade with *S. kratochvili (*= *Skrjabinotaenia* cf*. lobata* (C)) sequence (GU254058 + MG049969) from *A. flavicollis*, Bosnia and Herzegovina, but differed from this sequence by 2 SNPs.

One adult cestode from *M. agrestis* was initially identified microscopically as *Rodentolepis assymetrica* (formerly: *Hymenolepis assymetrica*). In 2022 this species was redescribed as *Kontrimavichusia assymetrica* (Makarikov and Binkienė, 2022), and therefore this species name has been assigned to our isolate. The 12S rDNA sequence of *K. assymetrica* grouped with a *Hymenolepis* sp. sequence from *M. arvalis* from Germany (Fig. 2) and displayed 98% similarity to it.

Three adult *Paranoplocephala* spp. cestodes from bank voles were genotyped, including also the *nad1* gene fragment, used for the delineation of new species from the *Paranoplocephala* complex by (Haukisalmi et al. 2014). In total four sequences were obtained, three for *cox1* and one for *nad1*. The *nad1* gene sequence showed the highest similarity (97%) to the sequence of *P. kalelai* from *C. rufocanus* from Russia (ON548176). On the phylogenetic tree this *Paranoplocephala* sequence grouped with a *P. kalelai* sequence from *C. rufocanus* from Finland (KJ778953) and constituted a sister group to *Paranoplocephala* (formerly: *Aprostatandrya) macrocephala* (Fig. 3). Three *cox1* gene sequences displaying the highest similarity (97%) to the sequence of *P. kalelai* from the small intestine of *C. glareolu*s, grouped on one branch with *P. kalelai* from *C. rufocanus* from Japan, reflecting also the close relationship to *P. omphalodes* (Fig. 1).

## Discussion

In the present study, several genetic markers were used for identification of cestode species recovered from wild rodents inhabiting the forests and open grasslands located in NE Poland. Genotyping and phylogenetic analyses allowed the identification of tapeworm species occurring in *C. glareolus, A. flavicollis, M. oeconomus* and *M. agrestis* from the Mazury Lake District. We demonstrated that among adult tapeworms, *C*. *henttoneni* is the dominant species in bank vole in this region of Poland, while among the larval forms *Mesocestoides* spp. dominated.

One of the main goals of our study was to identify cestodes from the *Paranoplocephala* complex based on a fragment of the mitochondrial *nad1* gene. Our results indicated the occurrence of only one species, *P. kalelai,* previously described from Finland, but known to exist in the North Western Palearctic (Haukisalmi et al 2007a). In Central and Eastern Siberia *P. buryatiensis* dominates in *C. rufucanus* (Haukislami et al. 2007b). Our isolate did not show any similarity to the newly described genera from the ‘*Paranoplocephala* complex’, i.e. *Tenoraia* or *Eurotaenia* g. n. (Haukisalmi et al. 2014). Therefore, since previous reports of species of the genus *Paranoplocephala* (*P. blanchardi* but now emended to *Microticola blanchardi)* (Behnke et al. 2001) (*P. omphalodes*) in Bajer et al. 2005; Behnke et al. 2008a, b; Grzybek et al. 2015 from bank voles from the Mazury Lake District were based entirely on morphological features, it is likely that some of these isolates could actually have been the currently molecularly identified species, *P. kalelai*. *Paranoplocephala kalelai* is also a significant member of the helminth community of *C. rufocanus* in Russia (Krivopalov et al. 2022). Phylogenetic analyses based on *nad1* and 12S *rDNA* clearly separated our sequences from the *P. omphalodes* sequences in GenBank. *Paranoplocephala kalelai* used to be common in bank voles in the Mazury lake district, with prevalence of 8.0% in 2000 (Bajer et al. 2005), although its prevalence has dropped significantly in recent years (Grzybek et al. 2015).

The prevalence of *C. henttoneni* in bank voles differed between our three study sites (highest at Urwitałt [24.3%] and Tałty (14.5%) but was relatively rare in Pilchy (6.7%) with four surveys at 3–4 year intervals combined). With sites combined, prevalence varied between the years with a peak prevalence in 2002 (25.1%) and lowest in 1999 (7.9%). For further breakdown by study site, year, host age and sex see (Behnke et al. 2001; 2008a, b; Grzybek et al. 2015). A recent complex genetic and phylogenetic study reported on the systematic position of *C. henttoneni* in the Catenotaeniid family (Haukisalmi et al. 2018). Both morphological and phylogenetic analyses of our isolates allowed for unambiguous identification of *C*. *henttoneni* in bank voles, which formed a separate, well-defined branch on phylogenetic trees. In the phylogenetic tree based on combined 28S rDNA and 12S-16S rDNA our *C. henttoneni* sequences formed a well-supported sister clade to *C. henttoneni* from *C. rutilus.* This cestode species has been regularly recorded in bank voles at our three forest study sites in the Mazury Lake District, ever since the beginning of our research in these locations in 1997 (Behnke et al. 2001, 2008a, b; Bajer et al. 2005; Grzybek et al. 2015*)*. Morphological features of this species accord entirely with the description of *C. henttoneni* (Haukisalmi and Tenora 1993; Haukisalmi et al. 2010- redescription]. Moreover, in the original description by Haukisalmi and Tenora (1993), *C. glareolus* was referred to as the type host for *C. henttoneni*, while *C. rutilus* was considered as an ‘other’ or incidental host. These authors recorded also some relatively small differences between the morphometrics of cestodes originating from bank voles and those from red backed voles, perhaps providing a basis for the minor difference we detected between our genetic sequences of worms from bank voles and the *C. henttoneni* sequence in GenBank of a Finnish isolate from *C. rutilus*. There is clearly a need for deeper molecular analyses building on the work of Haukisalmi et al (2018) who included 12S-16S sequences of *C. henttoneni* from Croatia, England and Sweden in their study. More sequences from bank voles and red backed voles from other European countries would help to establish whether *C. henttoneni* exists as host-related, or locally prevalent geography-related variants/subspecies.

The original description of *S. lobata* was from Africa (Baer 1925) and there exist also redescriptions of this species among African as well as European cestodes (Baylis 1927; Genov 1984). Tenora (1959) described the European form of *S. lobata* as *Catenotaenia (Spasskijela) kratochvili* Tenora 1959. In their comprehensive study of the molecular systematics and evolutionary history of the catentaeniids Haukisalmi et al (2018) proposed the existence of three distinct *Skrjabinotaenia lobata*-like species (A–C), of which one parasitizes mice of the genus *Apodemus* in Europe (*S. cf. lobata* C) and two (*S. cf. lobata* A and B) parasitize other murids in Africa. Accordingly, our combined 28S and 12S-16S rDNA sequences formed a separate clade from the African isolates, corresponding to *S. cf. lobata* species/genotype C (Fig. 4) The name *Spasskijela kratochvili* Tenora 1959 has been proposed for this European genotype of *S. lobata* (Haukisalmi et al. 2018). Based on their preliminary phylogenetic analyses Haukisalmi et al. (2018) reported that the genetic distances among European *S. lobata*-like isolates (sp. C/*S. kratochvili*) were minimal compared with interspecific distances, supporting the idea that there is a single wide-spread species (*Spasskijela kratochvili*) in Europe, parasitizing primarily mice of the genus *Apodemus*. However, no comparative morphological studies of these species have been conducted, and to-date no formal redescription has been published. Cestodes of this species have been regularly found in *Apodemus* spp. from the Mazury Lake District, constituting the dominant component of the helminth community with a prevalence of 27.4% (Bajer 2002 unpublished).

Based on morphology, one tapeworm from *M. agrestis* was identified as *Rodentolepis assymetrica* (formerly: *Hymenolepis assymetrica*). In 2022, this species was redescribed as *Kontrimavichusia assymetrica* (Makarikov and Binkienė, 2022), so this species name has been assigned to the tapeworm in the present study. *Kontrimavichusia assymetrica* has been previously found in *Microtus* spp. from the Mazury Lake District but was generally a rare species (prevalence < 1%) (Bajer 2002 unpublished).

Our previous studies were based on morphological identification of tapeworms (Behnke et al. 2001). However, molecular and phylogenetic analysis has enabled a more accurate identification of cestode species and genera, especially for larval cestodes. To the best of our knowledge, our data represent the first genetic identification of the species *P. kalelai* and *C. henttoneni* occurring in the bank vole in Poland and the first molecular typing of *S. cf. lobata* from *A. flavicollis*. The current study shows that the use of genetic and phylogenetic analysis allows for better, more accurate identification of tapeworms found in rodents, compared to the conventional methods based on morphological characters.

## Supplementary Information

Below is the link to the electronic supplementary material.

Supplementary file1 Cestodes: *Catenotaenia henttoneni* a. scolex; b. mature proglottids; c. uterus; *Spasskijela kratochvili *(*=Skrjabinotaenia* cf. *lobata* (C)) d. scolex; e. mature proglottids; f. uterus; *Paranoplocephala kalelai* g. scolex; h. mature proglottids; i. uterus; *Kontrimavichusia assymetrica* j. mature proglottids; k. uterus development; l. uterus; *Taenia polyacantha* m. larvae scolex; n. larvae hooks; *Mesocestoides melesi* o. larva; p. larval suckers; *Mesocestoides litteratus* r. larva; s. larval suckers (JPG 2627 KB)

Supplementary file2 (JPG 2125 KB)

Supplementary file3 Summary of tapeworm species, their geographical distribution, host species, genotyped gene fragments, and corresponding GenBank accession numbers (XLSX 13 KB)

Supplementary file4 (DOCX 20 KB)

## Data Availability

All relevant data is presented within manuscript files (text, tables, supplementary material); The datasets generated and analysed during the current study are available in the UW research data repository, 10.58132/YKN7IQ.
