# Supplementary figures and images for: Molecular identification of cestodes from rodents in the Mazury Lake District region of Poland
Source: Parasitol Res. 2026 Feb 16;125(1):26. doi: 10.1007/s00436-026-08629-x (PMC12909446; doi:10.1007/s00436-026-08629-x)

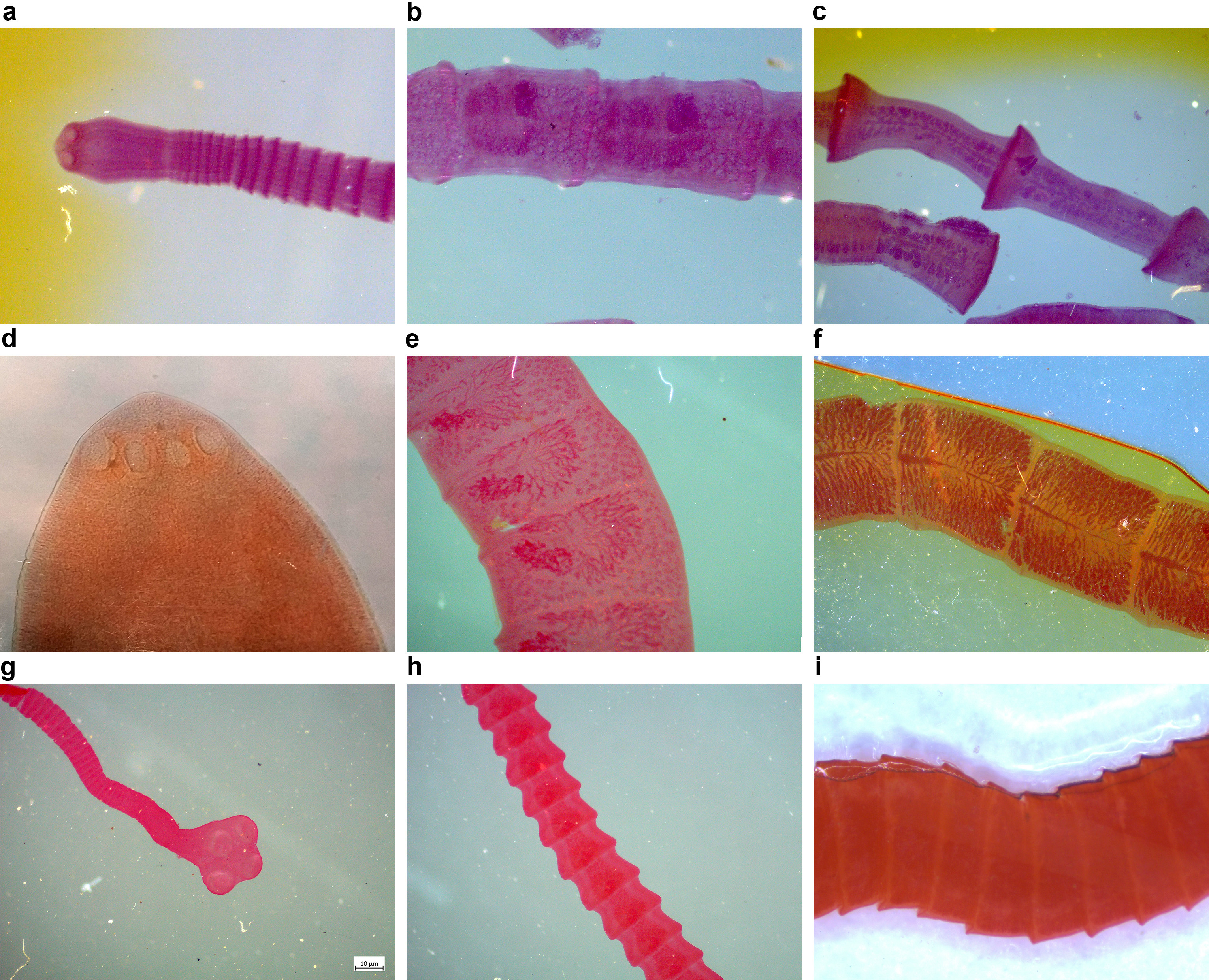

Supplement: Supplementary file 1 — Supplementary file1 Cestodes: Catenotaenia henttoneni a. scolex; b. mature proglottids; c. uterus; Spasskijela kratochvili (=Skrjabinotaenia cf. lobata (C)) d. scolex; e. mature proglottids; f. uterus; Paranoplocephala kalelai g. scolex; h. mature proglottids; i. uterus; Kontrimavichusia assymetrica j. mature proglottids; k. uterus development; l. uterus; Taenia polyacantha m. larvae scolex; n. larvae hooks; Mesocestoides melesi o. larva; p. larval suckers; Mesocestoides litteratus r. larva; s. larval suckers (JPG 2627 KB) [file 436_2026_8629_MOESM1_ESM.jpg]

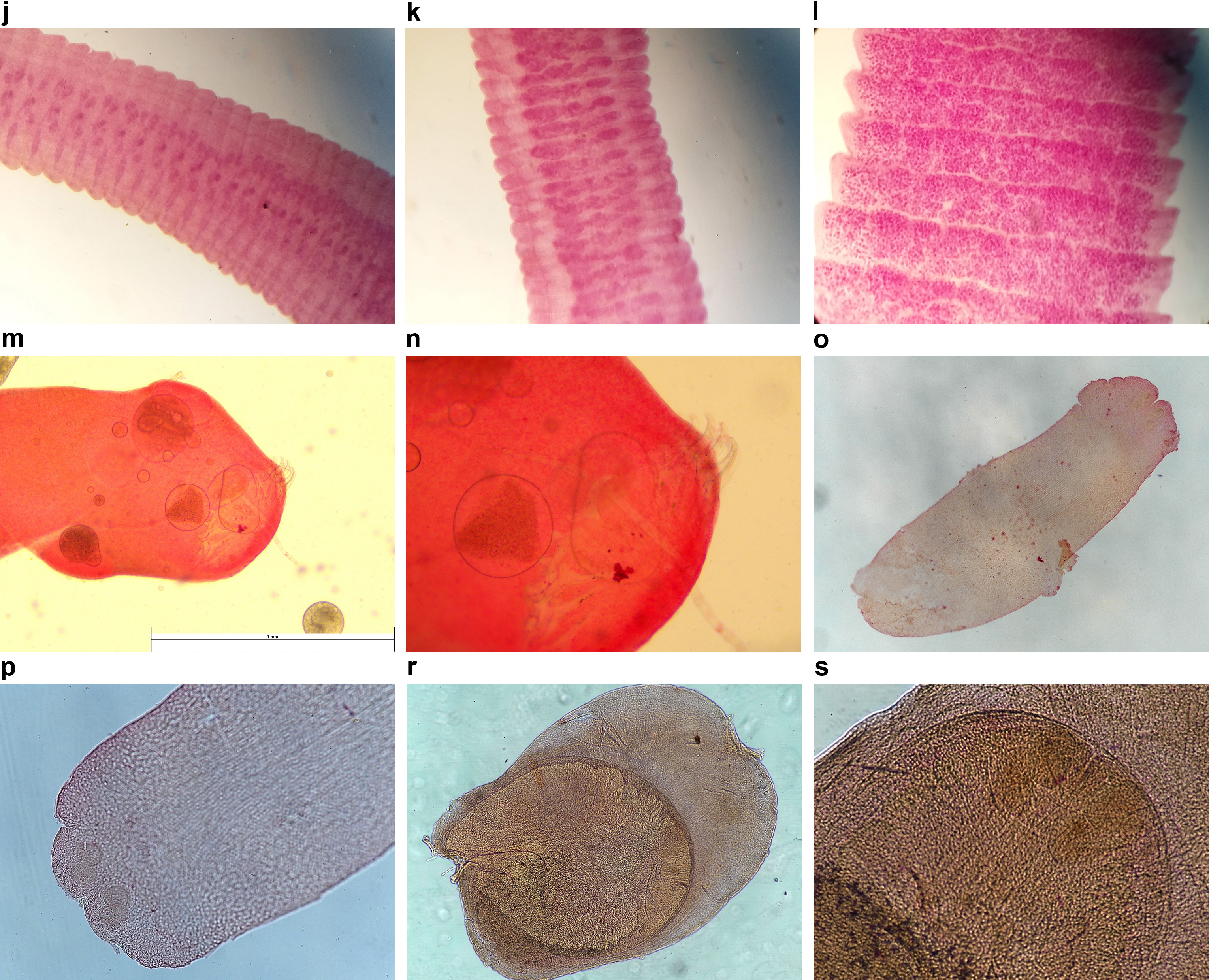

Supplement: Supplementary file 2 — Supplementary file2 (JPG 2125 KB) [file 436_2026_8629_MOESM2_ESM.jpg]
