# Supplementary material for: Molecular identification of cestodes from rodents in the Mazury Lake District region of Poland
Source: Parasitol Res. 2026 Feb 16;125(1):26. doi: 10.1007/s00436-026-08629-x (PMC12909446; doi:10.1007/s00436-026-08629-x)
Supplement: Supplementary file 4 — Supplementary file4 (DOCX 20 KB) [file 436_2026_8629_MOESM4_ESM.docx]

Additional file 1: Table S2. Tapeworm species used in the phylogenetic analyses of combined 12S-16S and 28S rDNA (Fig. 4), with their host species, geographical origin and accession numbers of nucleotide sequences. Family and subfamily of hosts (combinations of two capital letters in parentheses) shown after the specific name: CA, Cricetidae, Arvicolinae; CC, Cricetidae, Cricetinae; CN, Cricetidae, Neotominae; MG, Muridae, Gerbillinae; MM, Muridae, Murinae; NN, Nesomyidae, Nesomyinae; NC, Nesomyidae, Cricetomyinae; SS, Sciuridae, Sciurinae; SX, Sciuridae, Xerinae. Numbers in superscript indicate additional host species (see footnote). GenBank number for the first haplotype refers to the isolate used in phylogenetic analyses. New sequences indicated in bold.

|  |  |  |  |  |  |
| --- | --- | --- | --- | --- | --- |
| Tapeworm species | **Host species (family, subfamily)** | **Country, region** | **DNA code** | **28S GenBank number (haplotypes)** | **12S-16S GenBank number (haplotypes)** |
|  |  |  |  |  |  |
|  |  |  |  |  |  |
| *Catenotaenia apodemi* (Haukisalmi, Hardman & Henttonen, 2010) | *Apodemus peninsulae* (MM)^1^ | Russia, Buryatia | S26 | GU254052 | MG050000 |
| *C. cricetuli* (Haukisalmi, Hardman & Henttonen, 2010) | *Cricetulus barabensis* (CC) | Russia, Buryatia | W30 | GU254055 | MG049984 |
| *C. dendritica (*Goeze, 1782) | *Sciurus vulgaris* (SS) | Finland | 403 | GU254039 | MG049982 |
| *C. henttoneni* (Haukisalmi & Tenora, 1993) | *Myodes rutilus* (CA)^2^ | Finland | T28 | GU254054 | MG049995 |
| *C. microti* (Haukisalmi, Hardman & Henttonen, 2010) | *Microtus socialis* (CA) | Kazakhstan | AE4 | GU254040 | MG049991 |
| *C. pusilla* (Goeze, 1782) | *Mus musculus* (MM) | Czech Republic | EN6 | MG050008 | MG049952 |
| *C. peromysci* (Smith, 1954) | *Peromyscus californicus* (CN) | USA, California | C91 | GU443958 | MG049981 |
| *Catenotaenia* sp. A (Haukisalmi et al. 2018) | *Myodes californicus* (CA) | USA, Oregon | H52 | GU254050 | MG049986 |
| *Catenotaenia* sp. B (Haukisalmi et al. 2018) | *Craseomys rufocanus* (CA) | Japan, Hokkaido | JA317 | MG099778 | MG099780 |
| *Catenotaenioides kirgizica* (Tokobaev, 1959) | *Apodemus uralensis* (MM) | Kazakhstan | AE7 | GU254042 | MG049997 |
| *S.* cf. *lobata* A (Baer, 1925) | *Stenocephalemys albipes* (MM)^6^ | Ethiopia | EI8 | MG050040 | MG049973 |
| *S.* cf. *lobata* B | *Mastomys natalensis* (MM) | South Africa | FC2 | MG050038 | MG049974 |
| *S.* cf. *lobata* C | *Apodemus flavicollis* (MM)^7^ | Bosnia and Herzegovina | Z37 | GU254058 | MG049969 |
| *S. lucida* (Ortlepp, 1962) | *Aethomys chrysophilus* (MM*)*^8^ | South Africa | EH6 | MG050010 | MG049975 |
| *Skrjabinotaenia* sp. A | *Saccostomus campestris* (NC) | South Africa | EH9 | MG049979 | MG049979, MG049978 |
| *Skrjabinotaenia* sp. B | *Gerbilliscus brantsii* (MG) | South Africa | EO0 | MG050027 | MG049966 |
| *Skrjabinotaenia* sp. C | *Micaelamys namaquensis* (MM)^9^ | South Africa | EE7 | MG050016 | MG049955 |
| *Skrjabinotaenia* sp. D | *Rhabdomys dilectus* (MM) | South Africa | EF8 | MG050025 | MG049958 |
| *Skrjabinotaenia* sp. E | *Rhabdomys pumilio* (MM) | South Africa | FD0 | MG099777 | MG049961 |
| *Bertiella* sp. | *Rattus tanezumi* | Vietnam | DQ4 | MG099776 | MG049950 |
| *Catenotaenia henttoneni* | *Clethrionomys glareolus* (CA) | Poland  (this study) | 5-2023 | **PQ645089** | **PQ645079** |
| *Catenotaenia henttoneni* | *Clethrionomys glareolus* (CA) | Poland | 13-2022 | **PQ645090** | **PQ645080** |
| *Catenotaenia henttoneni* | *Clethrionomys glareolus* (CA) | Poland | 45-2018 | **PQ645091** | **PQ645081** |
| *Catenotaenia henttoneni* | *Clethrionomys glareolus* (CA) | Poland | 52-2018 | **PQ645092** | **PQ645082** |
| *Catenotaenia henttoneni* | *Clethrionomys glareolus* (CA) | Poland | 60-2014 | **PQ645093** | **PQ645083** |
| *Catenotaenia henttoneni* | *Clethrionomys glareolus* (CA) | Poland | 73-2018 | **PQ645094** | **PQ645084** |
| *Catenotaenia henttoneni* | *Clethrionomys glareolus* (CA) | Poland | 104-2018 | **PQ645095** | **PQ645085** |
| *Spasskijela kratochvili* | *Apodemus flavicollis* (MM) | Poland | SK6-2022 | **PQ645096** | **PQ645086** |
| *Spasskijela kratochvili* | Apodemus flavicollis (MM) | Poland | SK9-2022 | **PQ645097** | **PQ645087** |
| *Spasskijela kratochvili* | *Apodemus flavicollis* (MM) | Poland | SK14-2022 | **PQ645098** | **PQ645088** |

Additional host species based on molecular identification of cestodes: ^1^ *Apodemus uralensis* (MM); ^2^ *Clethrionomys* *glareolus* (CA); ^3^ *Myodes rutilus* (CA); ^4^ *Mastomys* *awashensis* (MM); ^5^ *Eliurus minor* (NN); ^6^ *Arvicanthis* sp. (MM); ^7^ *Apodemus sylvaticus* (MM); ^8^ *Micaelamys namaquensis* (MM); ^9^ *Mastomys natalensis* (MM).

Accession numbers for host vouchers (Museum of Southwestern Biology, University of New Mexico): ^10^ MSB:Mamm:155751, ^11^ MSB:Mamm:156267, ^12^ MSB:Mamm:155687.
